# Carbon‐Carbon Coupling on Inert Surfaces by Deposition of En Route Generated Aryl Radicals

**DOI:** 10.1002/anie.202010833

**Published:** 2020-10-29

**Authors:** Gianluca Galeotti, Massimo Fritton, Markus Lackinger

**Affiliations:** ^1^ Deutsches Museum Museumsinsel 1 80538 Munich Germany; ^2^ Department of Physics Technische Universität München James-Franck-Str. 1 85748 Garching Germany

**Keywords:** on-surface synthesis, radical coupling, scanning probe microscopy, surface chemistry

## Abstract

To facilitate C−C coupling in on‐surface synthesis on inert surfaces, we devised a radical deposition source (RDS) for the direct deposition of aryl radicals onto arbitrary substrates. Its core piece is a heated reactive drift tube through which halogenated precursors are deposited and en route converted into radicals. For the proof of concept we study 4,4′′‐diiodo‐*p*‐terphenyl (DITP) precursors on iodine‐passivated metal surfaces. Deposition with the RDS at room temperature results in highly regular structures comprised of mostly monomeric (terphenyl) or dimeric (sexiphenyl) biradicals. Mild heating activates progressive C−C coupling into more extended molecular wires. These structures are distinctly different from the self‐assemblies observed upon conventional deposition of intact DITP. Direct deposition of radicals renders substrate reactivity unnecessary, thereby paving the road for synthesis on application‐relevant inert surfaces.

## Graphical abstract

Terphenyl biradicals are generated from iodinated precursors en route by deposition through the reactive drift tube of a newly devised radical deposition source. Upon deposition onto iodine‐passivated metal surfaces, the terphenyl radicals self‐assemble into ordered structures but remain active for C−C coupling into dimers and extended oligomers.

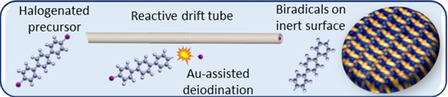

## Introduction

On‐surface synthesis is a maturing approach for the realization of extended covalent organic nanostructures that spark tremendous interest in fundamental science and await a promising future in applications.^[1]^ In terms of electronic π‐conjugation and overall stability, reactions that afford carbon‐carbon (C−C) coupling are of primary importance. In the established synthetic routes precursor molecules are deposited onto metal surfaces with accompanying or subsequent heating to thermally activate coupling.^[2]^ On‐surface C−C coupling was implemented by various reactions inspired from their solution analogues as Sonogashira,^[3]^ Glaser‐Hay,^[4]^ and vastly explored Ullmann‐type couplings^[5]^ as well as cyclo‐dehydrogenation.^[6]^ These reactions proceed exclusively on metal surfaces, whose chemically active contributions are required either for kinetic or thermodynamic reasons. Metal surfaces can lower reaction barriers and enhance reaction energies by stabilizing reaction intermediates, by‐products or final products.^[2]^ Alternatively, C−C coupling can be non‐thermally activated by light.^[7]^ Despite encouraging first results, the generality and utility of alternative activation schemes remains elusive.

Although metal surfaces are beneficial for characterization with electron‐based techniques such as scanning tunneling microscopy (STM) or X‐ray photoelectron spectroscopy (XPS), an impasse is reached when it comes to the fabrication of devices that exploit the unique electronic properties. Ultimately, this requires semiconducting or insulating substrates. Post‐synthetic decoupling by physically separating the nanostructures through an inert spacer layer represents a viable means for diminishing the influence of the metal surface. Proven strategies encompass intercalation,^[8]^ chemical transformation of the upper metal layers into an inert compound (e.g. silicides or oxides)^[9]^ or lateral manipulation on top of a posteriori deposited insulating thin films (mostly alkali halides).^[10]^ While decoupling facilitates characterisation of the nanostructure's inherent structures and electronic properties, it appears to be of limited use for actual device fabrication. Therefore, transferring the nanostructures from the growth to the target substrate remains the prevalent method.^[11]^ Yet, commonly applied template stripping is not only tedious,^[12]^ but is also carried out wet‐chemically, bearing high risks of compromising purity and integrity.

Accordingly, synthesis by direct C−C coupling on inert surfaces represents an important milestone. Only few successful examples of either inter‐ or intramolecular C−C coupling are reported on various non‐metallic surfaces as TiO_2_,^[13]^ calcite^[14]^ and alkali halides.^[15]^ Even though these examples represent major achievements, their specific requirements and conditions cannot alleviate the need for a facile and more general approach.

An intriguing concept is the direct deposition of activated species that can readily undergo C−C couplings on arbitrary surfaces. This idea was implemented by Gleason and co‐workers as “initiated Chemical Vapour Deposition—iCVD”. Thereby up to micron thick polymer films can be grown with the aid of directly deposited radicals that either polymerize themselves by addition reactions or initiate a polymerization in pre‐adsorbed monomers.^[16]^ For on‐surface synthesis, the temporal and spatial separation of activation and coupling removes the need for chemically active substrates, which could then be chosen for optimized coupling. The obvious activated species for C−C coupling are radicals. The utility of halogen‐substituents as leaving groups for thermal cleavage prior to deposition was already alluded in the seminal work by Grill et al.,^[5a]^ where they suggested the direct deposition of radicals for crucible temperatures above 590 K. Such an in‐crucible activation, however, does not allow sustainable deposition of radicals due to progressive polymerization of the evaporand. A more sophisticated scheme based on the separation of precursor sublimation, activation and deposition was proposed by Sakaguchi.^[17]^ They used a quartz tube reactor in a two‐zone furnace, where a flux of brominated precursors is generated by sublimation. Debromination is supposed to take place in the high temperature zone at the 625 K hot reactor walls. The supposedly created radicals are deposited downstream onto the target substrate in a lower temperature zone. Yet, in this work, conditions were applied where chemical activation is still feasible on the target substrate. The Au(111) surface was held at a temperature of 525 K, that is, high enough for debromination of intact precursors.^[18]^ Albeit this study proposes an intriguing concept, it could not provide unequivocal evidence for precursor activation prior to deposition.

We unambiguously demonstrate the generation of radicals from an iodinated precursor for subsequent deposition and C−C coupling on inert surfaces. As target substrates we have chosen Ag(111) and Au(111) passivated with chemisorbed iodine monolayers, because iodine passivation is facile, self‐limiting to one monolayer and renders the surface inert with respect to thermally activated dehalogenation.[^8^, ^19^] Iodine forms hexagonal √3×√3R±30° superstructures on both Au(111) and Ag(111) with similar lattice parameters of *a*=*b*=0.50 nm.^[20]^ More importantly, adsorbed aryl radicals form covalent‐like bonds with the chemisorbed iodine atoms,^[21]^ which may be helpful to stabilize smaller radical species for STM characterization. We used the 4,4′′‐diiodo‐*p*‐terphenyl (DITP) precursor as relatively simple and well‐studied model compound.^[22]^ Moreover, the targeted para‐poly‐phenylene (PPP) wires received significant interest,[^13a^, ^23^] not at least for the on‐surface synthesis of graphene nano‐ribbons by lateral fusion.^[22c]^ The en route generation of radicals was realized by deposition through a reactive drift tube. The resulting nanostructures were characterized by STM either directly as deposited or after subsequent mild heating. As an important control, results are compared to experiments with conventional deposition of intact DITP.

## Results and Discussion

Terphenyl biradicals were generated and deposited with a dedicated radical deposition source (RDS, see SI section 2). Its core piece is a heated drift tube comprised of gold‐plated stainless steel through which the precursors are indirectly deposited. While on gold surfaces deiodination readily occurs at room temperature,[^18^, ^24^] a sufficiently high drift tube temperature of ≈500 K was required to facilitate passage of en route generated radicals by sequences of adsorption‐desorption processes.

**Figure 1:**
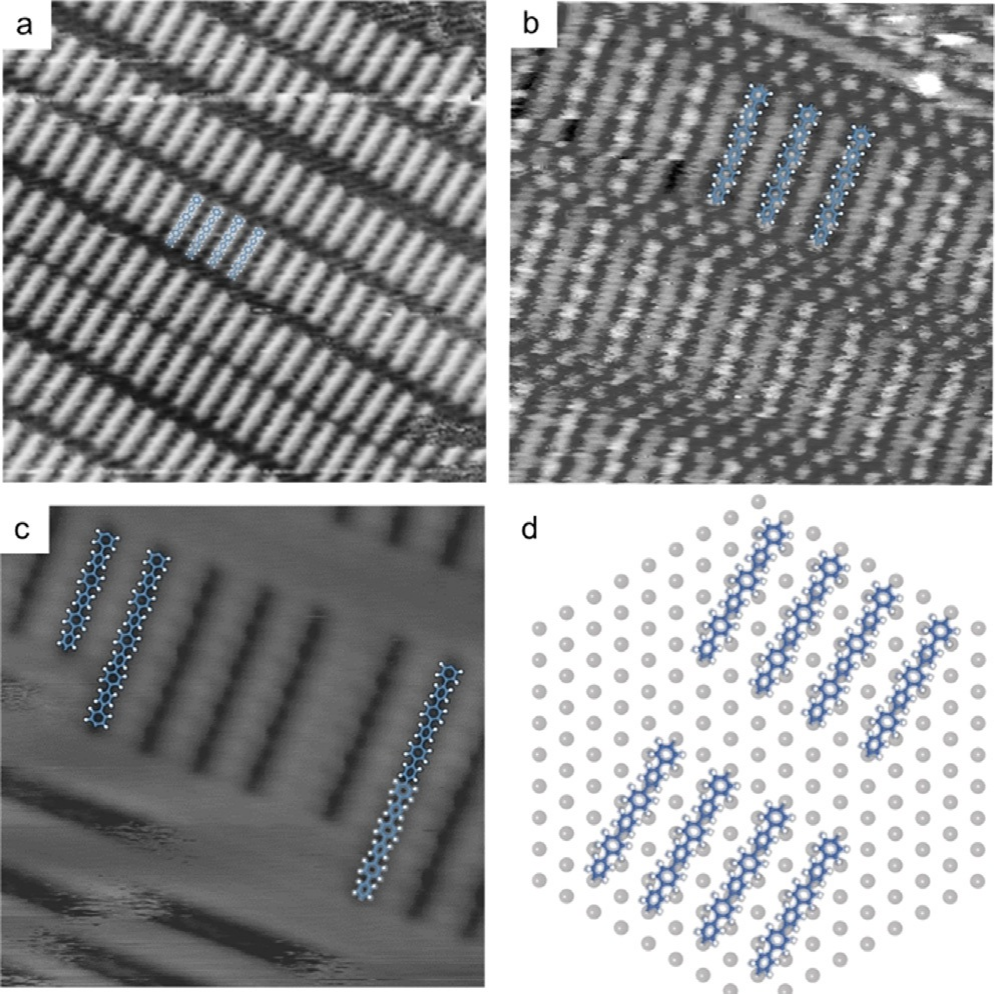
RDS deposition of biradicals from DITP precursors onto I‐Au(111) held at room temperature. a)–c) STM images acquired at room temperature showing regular structures of rod shaped entities; Note that the image in c) exhibits an occasionally observed inverted contrast (adsorbed biradicals appear with lower height); (image parameters: a: 25×25 nm^2^, 108 pA, 0.60 V; b: 10×10 nm^2^, 64 pA, 1.49 V; c: 10×10 nm^2^, 104 pA, −0.96 V) d) tentative model based on the experimentally deduced orientation of sexiphenyl with its long axis aligned along the <11> directions of the iodine monolayer (grey spheres, the underlying metal surface is omitted for clarity).

STM images acquired after deposition with the RDS onto iodinated metal surfaces held at room temperature are presented in Figure 1. The overview image unveils a highly regular arrangement of rod‐shaped entities, organized in unevenly spaced lamellas. The (2.5±0.1) nm long rods clearly exceed the dimensions of DITP precursors (1.58 nm from iodine to iodine, cf. SI, Figure S2). But their length is consistent with C−C bonded terphenyl dimers, that is, sexiphenyl, as demonstrated by the overlay, and further corroborated by the internal STM contrast showing six protrusions, corresponding to the phenyl rings (Figure 1 b). Moreover, we occasionally observed longer entities that are consistent with C−C bonded terphenyl trimers ((3.7±0.1) nm) or tetramers ((5.0±0.1) nm) (Figure 1 c). The chemical nature of the termini, however, remains nonspecific. Yet, we can already exclude remaining iodine substituents, simply because there is not enough space to accommodate them for the narrowest observed lamella spacings.

**Figure 2:**
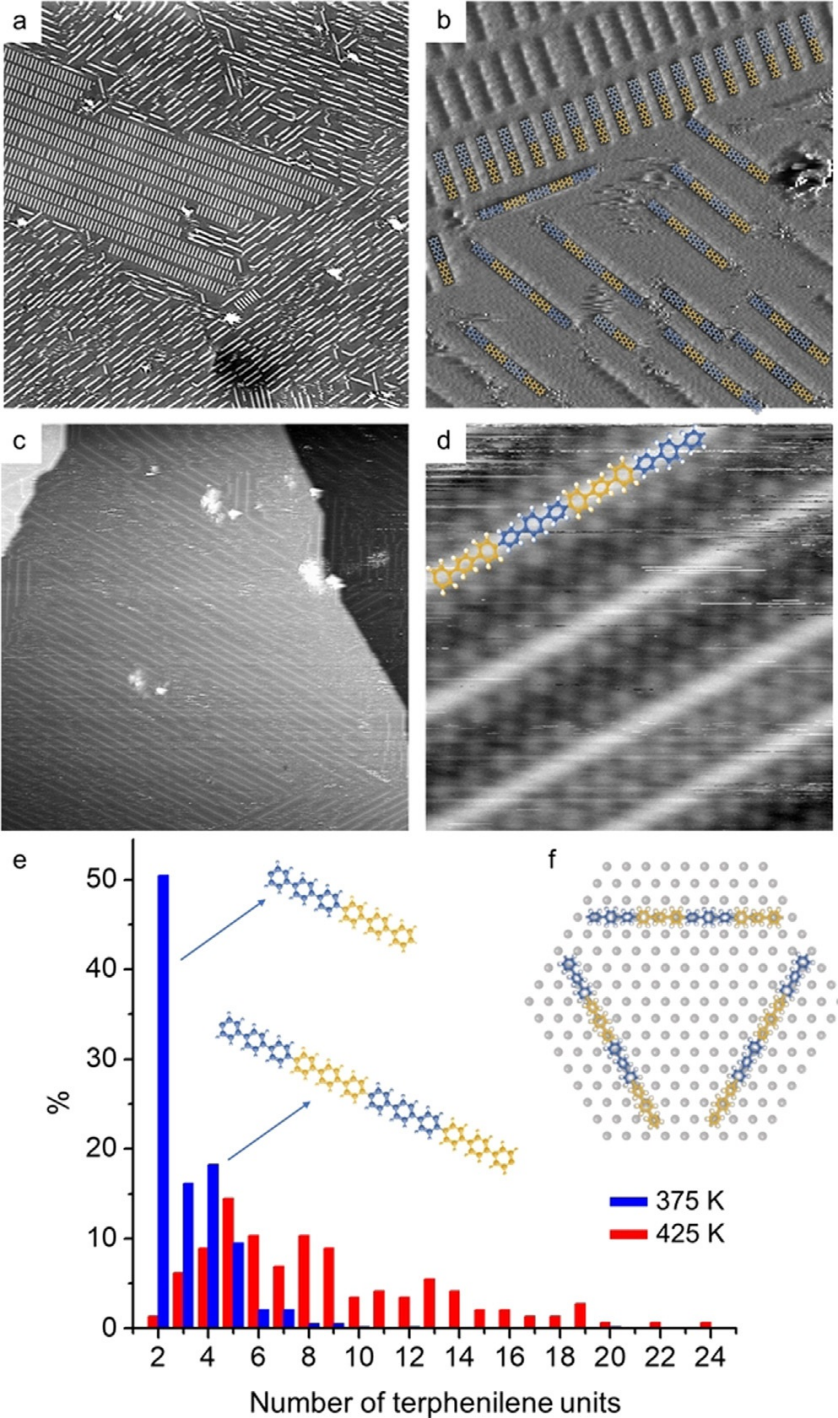
RDS deposition of biradicals from DITP precursors onto I‐Au(111) held at room temperature. STM images acquired after subsequent annealing to a), b) 375 K and c), d) 425 K; (image parameters: a: 120×120 nm^2^, 42 pA, 1.76 V; b: 30×30 nm^2^, 104 pA, −0.96 V; c: 100×100 nm^2^, 84 pA, −1.19 V; d: 8×8 nm^2^, 101 pA, −0.11 V). e) Histograms of the oligomer length distribution after heating to 375 K (blue, 2272 terphenyl moieties counted) and 425 K (red, 1254 terphenyl moieties counted) f) Ball and stick model highlighting the <11> orientation of the oligomers with respect to the iodine monolayer; C atoms in the oligomers are alternatingly colored in blue and yellow to highlight the terphenyl moieties, H in white, I in grey.

Accordingly, we propose that the rods correspond to biradicals that are stabilized by covalent‐like bonds formed at the termini with chemisorbed iodine atoms in the monolayer.^[21]^ The model in Figure 1 d suggests an excellent geometric match. Thereby strain is minimized, resulting in relatively strong adsorption of the comparatively small sexiphenyl biradicals that facilitates sufficient stability for room temperature STM imaging. Despite the covalent bonding with the iodine monolayer, the adsorbed biradicals still remain active for progressive C−C coupling. The STM image in Figure 2 a was acquired after subsequent mild heating to 375 K. It shows a remaining domain of sexiphenyl biradicals, but also corroborates the formation of more extended linear oligomers. These are organized in domains with uniform alignment along the three equivalent <10> directions of the iodine monolayer. Again the oligomer lengths are quantized in terphenyl units as illustrated by the overlays (Figure 2 b). Statistical analysis of the length distribution of a sample heated at 375 K reveals ≈50 % dimers and <5 % of oligomers longer than five terphenyl units (Figure 2 e). Higher heating at 425 K profoundly alters the length distribution. Only a small fraction of dimers remains, while most of the oligomers (>80 %) are comprised of five up to 24 terphenyl units (Figure 2 e). Apparently, heating provides additional thermal energy to overcome barriers that prevent further coupling at room temperature. These barriers arise from the necessity of breaking radical‐iodine bonds to facilitate diffusion and C−C bond formation. Even though longer wires are sufficiently stable to be imaged at room temperature, we did not resolve individual phenyl rings, in accord with previous studies of short PPP chains on copper surfaces.^[25]^ This is consistent with a dominating stabilization on the iodine passivated surfaces by covalent anchoring at the termini, which is comparatively strong for sexiphenyl, but too short‐ranged for longer wires.

**Figure 3:**
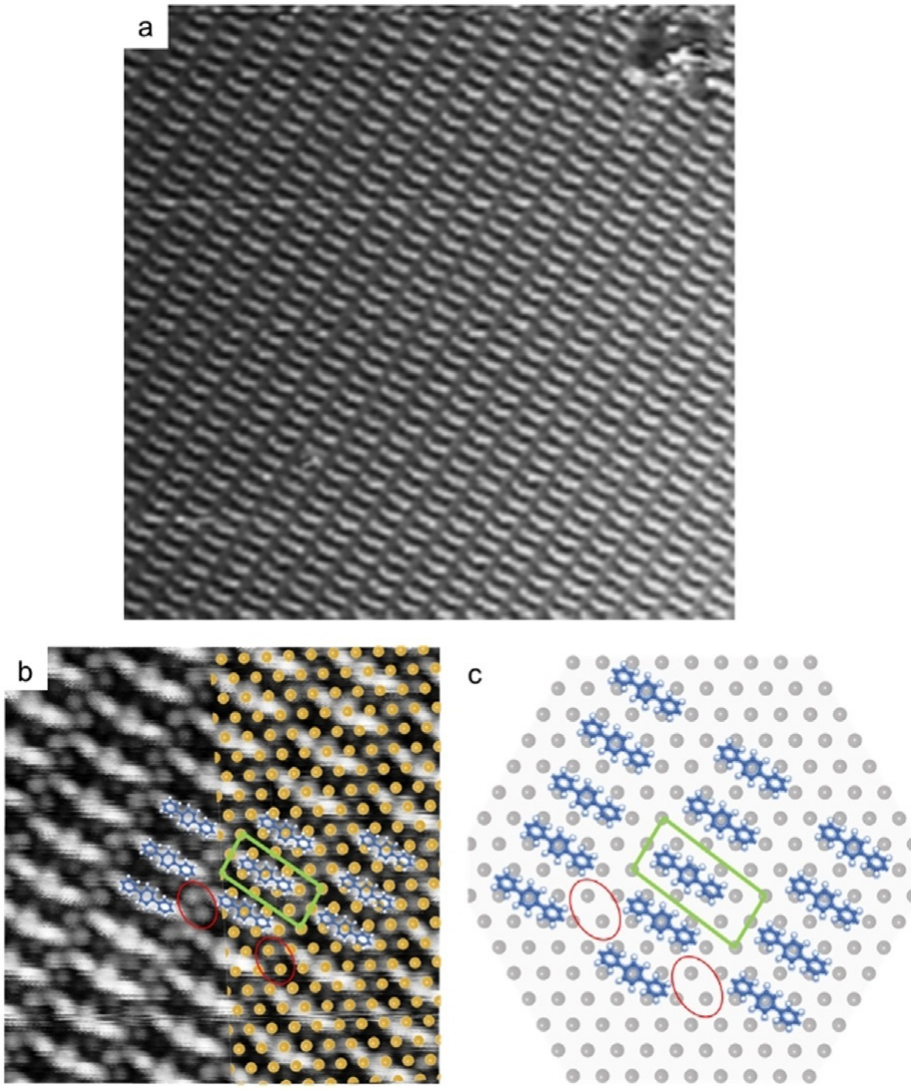
RDS deposition of biradicals from DITP precursors onto I‐Ag(111) held at room temperature. The sample was immediately transferred into the cold STM and images were acquired at ≈85 K. a) overview and b) close‐up showing a regular arrangement of rods assigned as terphenyl biradicals (a: 30×30 nm^2^, 16.5 pA, 1.52 V; b: 9×9 nm^2^, 17.5 pA, 1.55 V). c) tentative model based on the commensurate unit cell; red cycles serve as visual aids for comparison with the STM image.

In a further series of experiments, samples were immediately cooled to ≈85 K after room temperature deposition with the RDS. STM images acquired at low temperatures similarly show a highly regular arrangement of rods with domain sizes up to 100 nm. The self‐assembly is periodic, hence a primitive unit cell can be assigned with lattice parameters *a*=(2.1±0.1) nm, *b*=(1.0±0.1) nm, *γ*=96° ±3° that agree well with a commensurate (3 5; 2 0) superstructure with respect to the iodine lattice. Noteworthy, also for this preparation some dimers were occasionally observed (see SI, Figure S3). Yet, the vast majority of rods are only (1.2±0.1) nm long, that is, significantly shorter as compared to those in Figure 1. The rods also appear with appreciable internal STM contrast, featuring two smaller bright protrusions at its termini and a less bright larger feature at the center. The measured (0.42±0.05) nm spacing between the protrusions agrees well with the phenyl‐phenyl distance of 0.44 nm in terphenyl. These observations are consistent with identifying the shorter rods as terphenyl biradicals as further illustrated by the overlay in Figure 3 b. The model in Figure 3 c suggests a less favorable size match between terphenyl biradical and iodine monolayer. Consequently, surface bonding of the smaller biradical strains the terphenyl backbone, resulting in a lower stability as compared to adsorption of sexiphenyl biradicals. This also explains why terphenyl biradicals are only observed when samples are instantaneously quenched.

The experiments with immediate sample cooling confirm the deposition of terphenyl biradicals. To also corroborate generation of the deposited radicals by the RDS, control experiments were carried out with conventional deposition of intact DITP onto iodinated metal surfaces.

**Figure 4:**
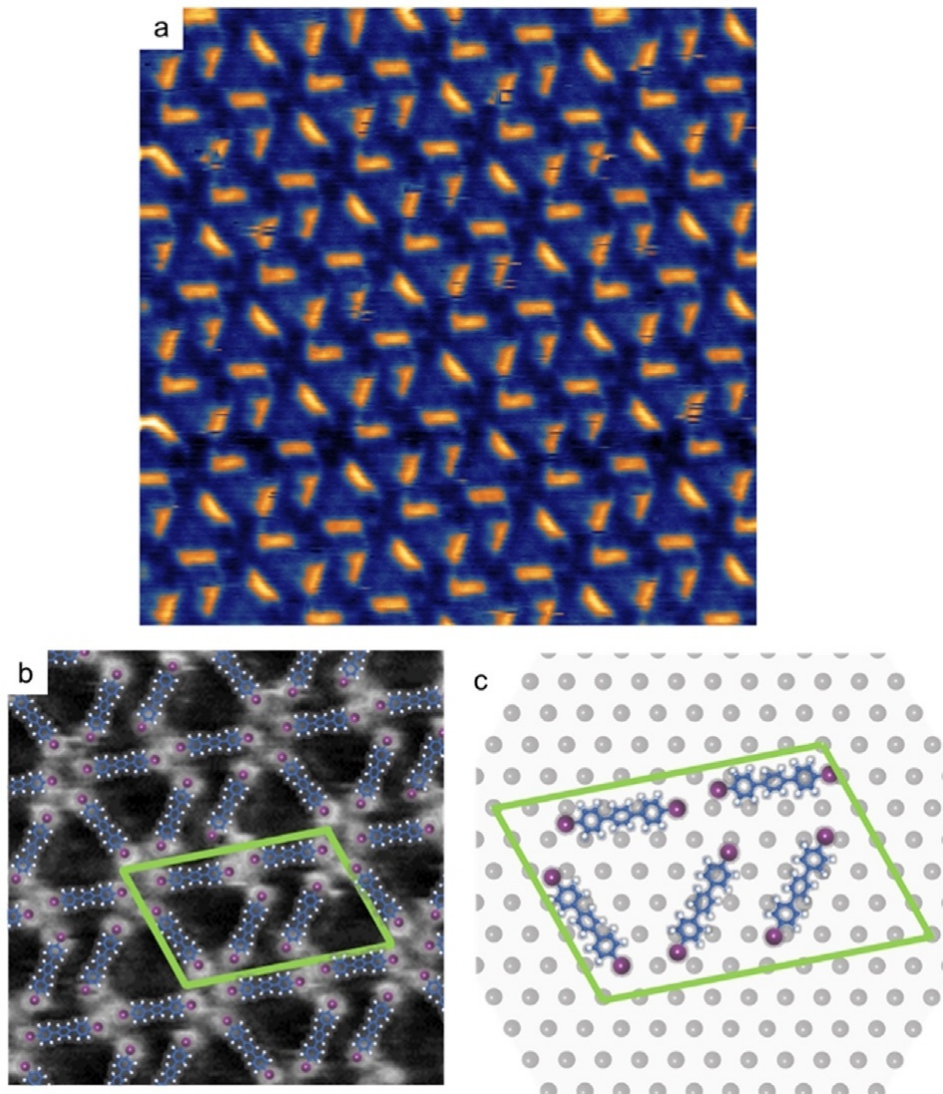
Conventional deposition of intact DITP molecules onto I‐Ag(111) held at room temperature. STM images showing the a) normally and b) occasionally observed contrast, which highlights the iodine‐substituents; (image parameters: a: 20×20 nm^2^, 72 pA, −1.21 V; b: 10×10 nm^2^, 73 pA, −1.21 V) c) tentative model derived from the STM images. The self‐assembly is stabilized by two distinct binding motifs either exclusively based on halogen‐halogen bonds or a combination with halogen‐hydrogen bonds (see SI, section 5).

STM images of a typically observed highly ordered structure are shown in Figure 4, but other polymorphs were similarly observed (see SI, section 5). Most importantly, all structures are supramolecular assemblies of intact DITP. Heating the DITP self‐assemblies on I‐Ag(111) to 425 K resulted in almost full desorption of DITP, leaving a pristine surface behind. On the one hand, this confirms the inertness of I‐Ag(111) for dehalogenation. On the other hand, it provides further evidence that dehalogenation is indeed achieved by the RDS. We occasionally observed indications of linear oligomers at step‐edges, suggesting some deiodination at sparse reactive sites (see SI, Figure S6). Also incompletely iodine passivated surfaces exhibit some remaining reactivity (see SI, section 7)

## Conclusion

In summary, we demonstrate the feasibility of direct deposition of radicals generated from the iodinated DITP precursor en route in a reactive drift tube. Its hot gold surface catalyzes the dissociation of iodine‐substituents from DITP precursors and conducts the generated biradicals. STM images acquired after deposition onto iodinated metal surfaces reveal regular arrangements of terphenyl biradicals that spontaneously dimerize into sexiphenyl biradicals at room temperature. Based on this observation, we propose an important role of the registry between biradical species and iodine monolayer for their surface stability. Control experiments with conventional DITP deposition demonstrate that I‐Ag(111) surfaces are highly inert with respect to dehalogenation, and also corroborate generation of radicals by the RDS for downstream deposition. The adsorbed species maintain their radical character as confirmed by their ability to further couple into extended linear structures upon mild heating. Accordingly, iodine passivated metal surfaces proved ideal for initial RDS studies as they offer a unique combination of inertness for dehalogenation and stickiness for the adsorption and stabilization of radical species. Among thousands of coupled terphenyl units only a few structures were observed that may be non‐linear junctions (see SI, Figure S8). Given that those could still arise from isomer impurities in the precursor, this observation suggests that the RDS does not induce 1,2‐rearrangements, where the radical site migrates to the adjacent carbon atom. This is important good news for using the RDS in the reticular synthesis of covalent nanostructures, because it implies that the sites for C−C coupling are fully predetermined by the precursor's halogen‐substitution pattern. However, a possible favourable role of the covalent bonding between radicals and iodinated surface remains to be explored.

Successful implementation of the RDS is our base camp for further exploring on‐surface synthesis with radicals. Exciting future experiment could tackle more abundant brominated or more complex precursors for targeting two‐dimensional polymers. Moreover, more fragile precursors could by tested, as for instance molecules with sulfur‐containing heterocycles that are key components in molecular electronics,^[28]^ but unfortunately prone to on‐surface decomposition.^[29]^ An obvious extension would be the use of alternative surfaces, where the RDS facilitates thus far elusive studies of deposition, diffusion and coupling of radicals at arbitrary temperatures.

## Conflict of interest

The authors declare no conflict of interest.

## Supporting information

As a service to our authors and readers, this journal provides supporting information supplied by the authors. Such materials are peer reviewed and may be re‐organized for online delivery, but are not copy‐edited or typeset. Technical support issues arising from supporting information (other than missing files) should be addressed to the authors.

Supplementary
