# Supplementary material for: Carbon‐Carbon Coupling on Inert Surfaces by Deposition of En Route Generated Aryl Radicals
Source: Angew Chem Int Ed Engl. 2020 Oct 29;59(50):22785–9. doi: 10.1002/anie.202010833 (PMC7814669; doi:10.1002/anie.202010833)
Supplement: Supplementary file 1 — Supplementary [file ANIE-59-22785-s001.pdf]

Angewandte Chemie
Eine Zeitschrift der Gesellschaft Deutscher Chemiker

Supporting Information

Carbon-Carbon Coupling on Inert Surfaces by Deposition of En Route Generated Aryl Radicals

*Gianluca Galeotti, Massimo Fritton, and Markus Lackinger**

anie_202010833_sm_miscellaneous_information.pdf

Supporting Information

1. Materials and Methods

Sample preparation and characterization were carried out in ultra-high vacuum at a base pressure $<3\times10^{-10}$ mbar. Ag(111) and Au(111) single crystal surfaces and thin Au(111) films on mica (Georg Albert PVD - Beschichtungen) were prepared by cycles of Ar^+-ion sputtering at 500 eV for 10 min followed by annealing at 773 K for 15 min. The 4,4″-diiodo-*p*-terphenyl (DITP) precursor was purchased from TCI with a purity of > 98%. For iodine passivation freshly prepared metal surfaces were exposed to 1×10^{-7} mbar (Ag) or 5×10^{-7} mbar (Au) of I_2 vapor for 10 min in a separate preparation chamber and subsequently heated to 423 K for 15 min. Successful preparation of a closed iodine monolayer was verified by atomically resolved STM imaging prior to further use. Radiative heaters were used for sample heating with reported temperatures read out by a type K-thermocouple firmly attached adjacent to the sample. Deposition of DITP through the RDS was carried out with a crucible temperature of 428 K for (30…45) min, whereas for conventional deposition of DITP a crucible temperature of 408 K and a deposition time of (45…60) min were used. In both cases, the iodinated metal surfaces were held at RT during deposition. The drift tube of the RDS was cleaned by annealing at 750 K for 30 min directly before deposition to desorb possibly remaining iodine from its active gold surface. The RDS was allowed to thermalize at its operation temperature for at least 20 min before deposition.

STM images were acquired in constant current mode either at room temperature or low temperature (~85 K) using home-built instruments operated by a SPM 100 controller from RHK. Electrochemically etched tungsten tips were used and in-situ conditioned by Ar^+-ion sputtering (at 1.3 keV kinetic energy and +150 V counter voltage applied to the tip). All bias voltages are referred to the tip. STM images were processed by plane levelling using the Gwyddion software.[1]

2. Radical Deposition Source (RDS)

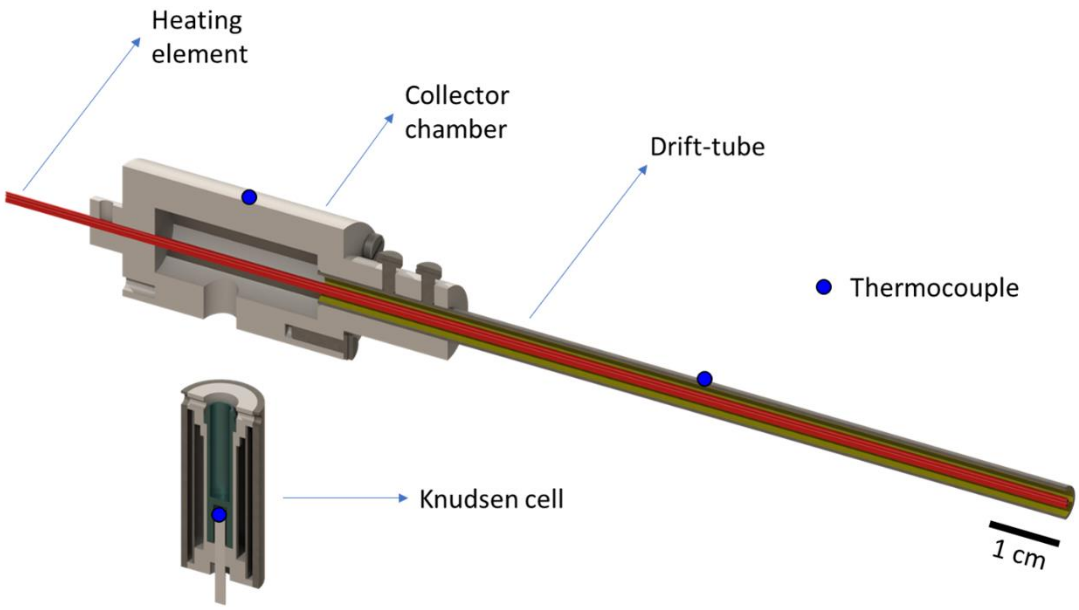

Figure S1. Cross-sectional view of the Radical Deposition Source including the Knudsen cell.

A sketch of the RDS is shown in Figure S1. The precursor molecules, DITP in this case, are evaporated with a conventional Knudsen cell into a reactive drift tube comprised of gold-plated stainless steel. Precursor activation by deiodination occurs on the hot walls of the drift tube. Although its gold surface should catalyze deiodination already at room temperature,[2] elevated tube temperatures are required to facilitate passage of en route generated radicals by sequences of adsorption-desorption processes. Precursors are deposited out of line of sight with the sample to avoid deposition of incompletely activated species, i.e. a contamination that would inevitably result in defect formation. This is achieved by means of an inert collector chamber made from Macor that also efficiently guides the molecules into the drift tube. It is independently heatable by means of a wrapped around heating wire. Moreover, the collector chamber serves as a mechanical fixture for the drift tube and its heating element. The drift tube is radiatively heated from the inside at its full length by an axially aligned filament that is encapsulated in an alumina capillary to avoid direct contact with the precursor. On the one hand heating the drift tube from the inside is highly efficient, on the other hand blocking the center of the drift tube by the heating element enhances the probability of molecular collisions with the tube wall. Both the alumina capillary used as encapsulation of the heating element and the Macor of the collector chamber should be inert with regard to a possible deiodination on its surface at the respective operation temperatures.

The RDS facilitates nearly independent control of three temperatures: (1) crucible of the Knudsen cell; (2) collector chamber; (3) drift tube; In the proposed design, the drift tube heating element also partially accounts for heating of the collector chamber. The crucible temperature controls the overall deposition rate of radicals. The temperature of the collector chamber is maintained (5..10) K above the respective crucible temperature to avoid condensation of molecules. The drift tube temperature has to be sufficiently high to facilitate dehalogenation and desorption of the (partly) dehalogenated species. Yet, the drift tube temperature (measured with a thermocouple at its outer surface) must not be too high to jeopardize the integrity of the molecular backbone.

3. Length comparison

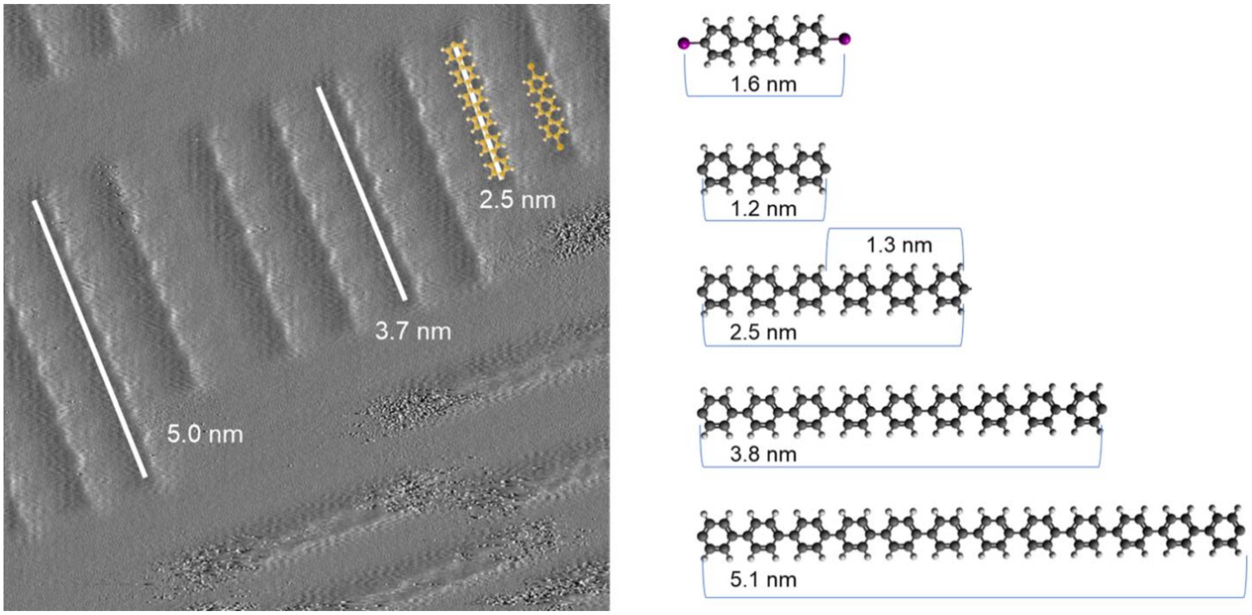

Figure S2. STM image acquired after RDS deposition onto I-Au(111) at room temperature (image parameters: 10×10 nm^2, 104 pA, -0.96 V). The lengths of the observed rods are quantized in terphenyl units, i.e. consistent with C-C bonded terphenyl dimers, trimers and tetramers as shown on the right hand side. None of the observed lengths match with that of the intact DITP precursors.

4. Terphenyl dimers and oligomers on I-Ag(111)

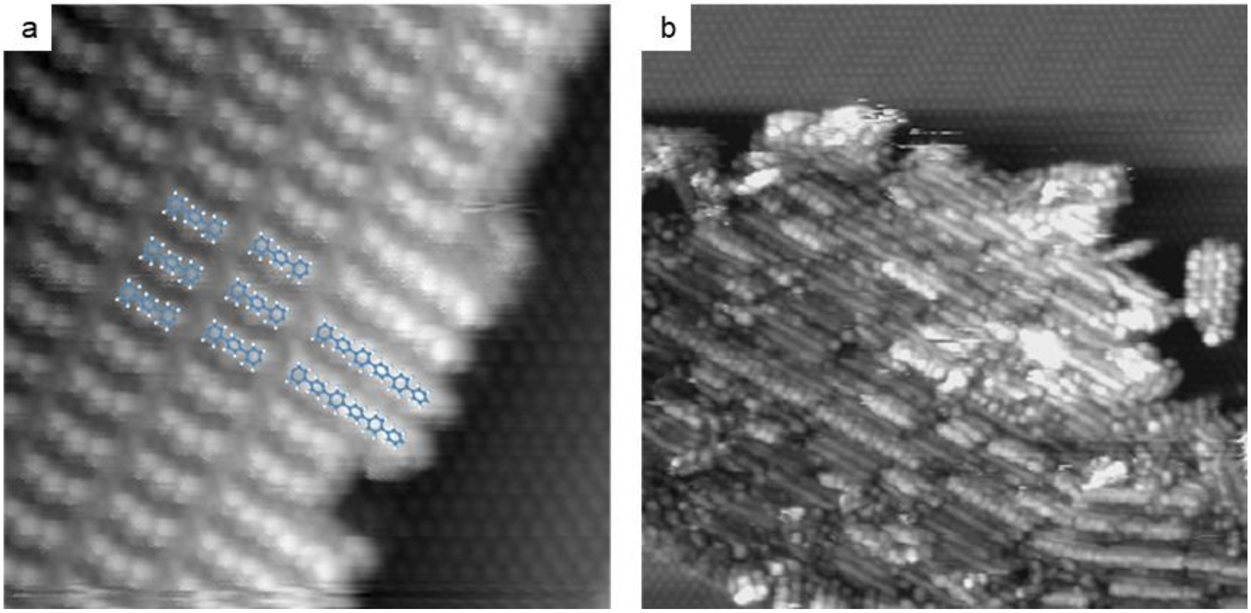

Figure S3. RDS deposition of biradicals from DITP precursors onto I-Ag(111) held at room temperature. The sample was immediately transferred into the cold STM after deposition. **a)** STM image acquired directly after cooling (image parameters: 10×10 nm^2, 18 pA, 1.52 V); Apart from the prevalent terphenyl biradicals, we occasionally observed covalent dimers, i.e. sexiphenyl biradicals at the domain edges. The iodine monolayer accounts for the background. Interestingly, these sexiphenyl biradicals are aligned along the <11> directions, whereas the terphenyl biradicals feature a slightly tilted orientation; **b)** STM image acquired at low temperature after the sample of a) was annealed to 675 K (image parameters: 20×20 nm^2, 13 pA, 1.64 V). Heating at this relatively high temperature results in the formation of linear wires, but also shows the onset of degradation. The iodine monolayer in the upper part features a height modulation that can be assigned to a reconstruction associated with a uniaxial compression of the iodine lattice.[3]

5. Self-assembly of intact DITP

Deposition of intact DITP with a conventional Knudsen cell onto iodine passivated surfaces resulted in the formation of various self-assembled phases (see Figure 4 of the main manuscript and Figures S4 and S5). While we were not able to selectively steer formation of a distinct polymorph, it is important to note that all structures observed upon conventional deposition of DITP are well explained as supramolecular assemblies of intact molecules. More importantly, these structures are vastly different from those observed upon deposition with the RDS (see Figures 1 and 2 of the main manuscript), providing further evidence for deposition of a chemically distinct species with the RDS, i.e. the dehalogenated terphenyl biradical.

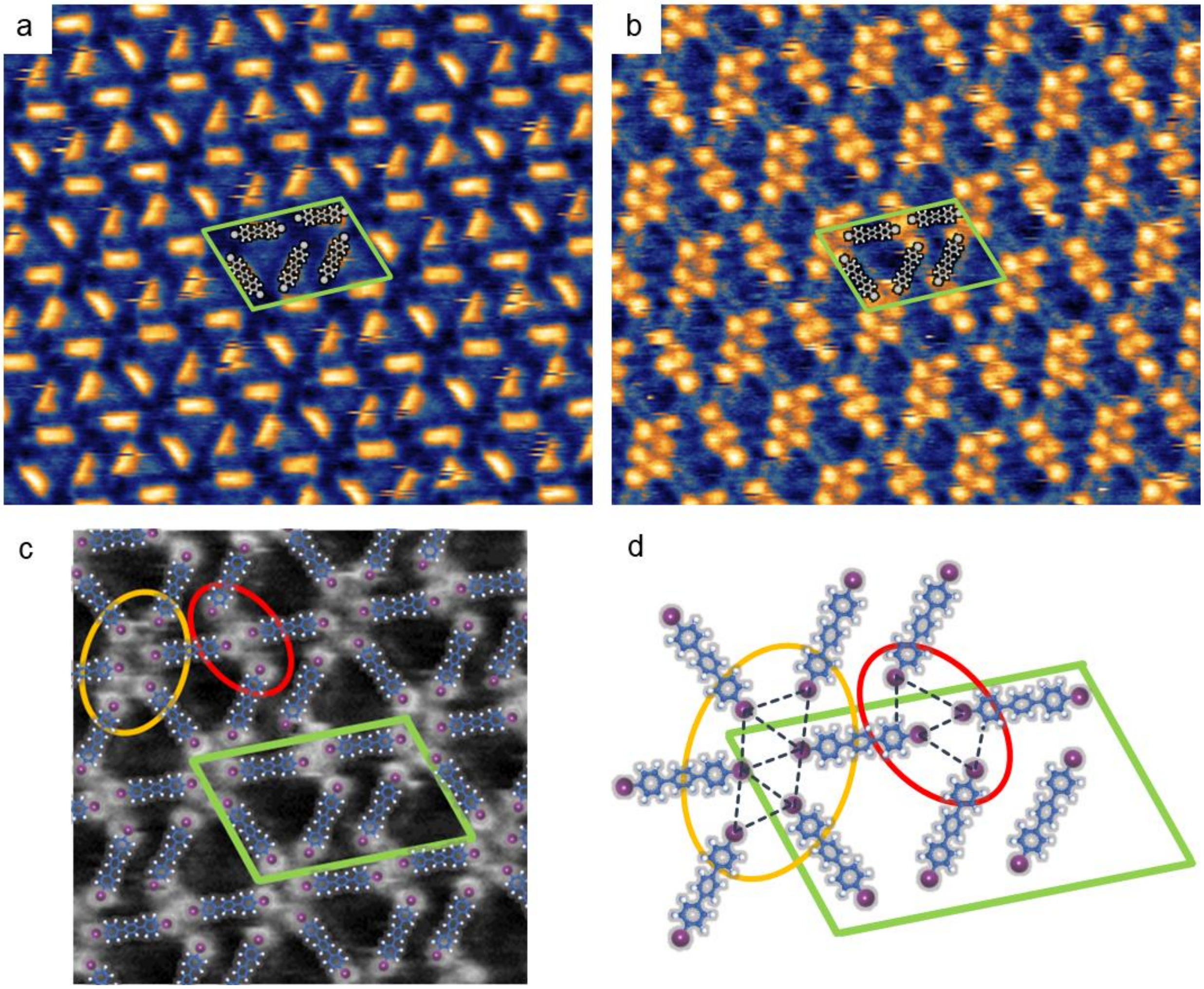

Figure S4. Conventional deposition of intact DITP molecules onto I-Au(111) held at room temperature. Sequentially acquired STM images showing the two observed different contrasts with **a)** terphenyl backbones and **b)** iodine-substituents highlighted. The overlays show the molecular arrangement within a unit cell. Since similar image parameters were used (19×16 nm^2, 72 pA, -1.21 V), the contrast change is attributed to a spontaneous tip change. **c)** close-up of b) with superimposed molecular model; **d)** detailed view of the molecular model with markers of the unit cell and the two different binding motifs corresponding to the STM image in c);

The self-assembly of intact DITP shown in Figure S4 features a relatively large unit cell with lattice parameters a=(4.7 ± 0.1) nm, b=(3.0 ± 0.1) nm and γ=110° ± 3° corresponding to a commensurate $\begin{pmatrix} 10 & 2 \\ 0 & 6 \end{pmatrix}$ superstructure with respect to iodine. Apart from the rod-like appearance of DITP in Figure S4a, we occasionally observed a different contrast, shown in Figure S4b, that brings out the iodine-substituents (see close-up in Figure S4c). This not only indicates deposition of intact DITP, but also helps to derive the structural model presented in Figure S4d. The molecular arrangement suggests stabilization by both intermolecular halogen-halogen and halogen-hydrogen bonds (as indicated by the dashed lines),[4] which is the prevalent binding in the self-assembly of halogenated tectons.[5] This structure features two distinct binding motifs highlighted by orange and red ellipses in Figures S4c and S4d. The orange marked binding motif is dominated by halogen-halogen interactions between all six iodine-substituents with arrangements reflecting the X_3 synthon common for aryl halides.[6] The red marked binding motif features both halogen-halogen and halogen-hydrogen interactions, and was similarly reported in the literature.[7]

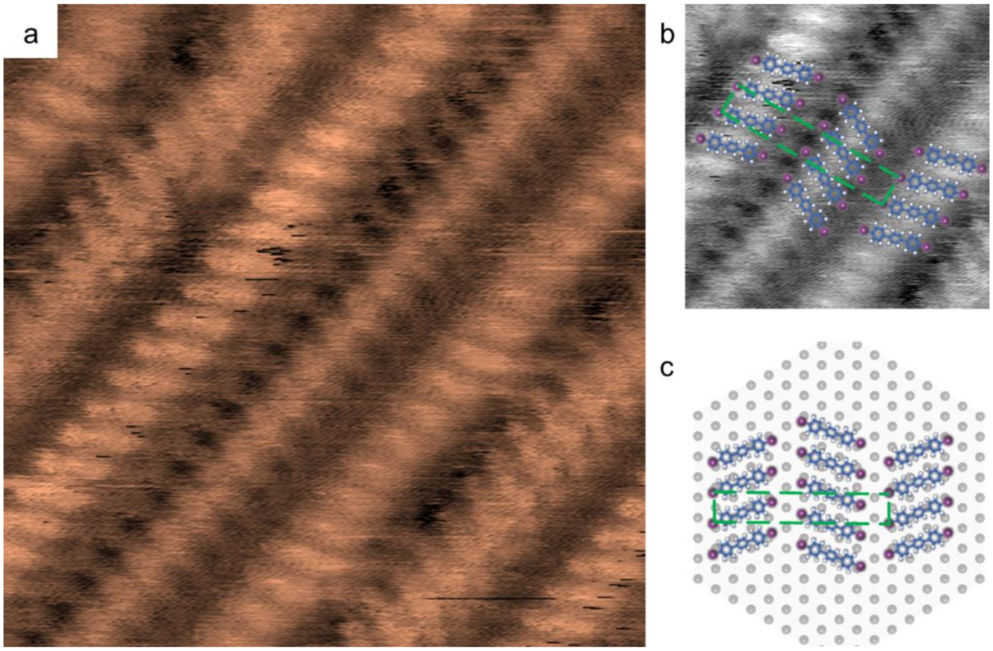

Figure S5. Conventional deposition of intact DITP molecules onto I-Au(111) held at room temperature. This alternative lamella polymorph was observed on both I-Ag(111) and I-Au(111) surfaces. **a)** STM image of the lamella polymorph and **b)** close-up with molecular overlay (image parameters: a: 15×15 nm^2, 107 pA, 0.96 V; b: 9×9 nm^2, close up of a); **c)** Tentative model of the lamella polymorph suggesting a joint stabilization by intermolecular halogen-halogen bonds and molecule-surface interactions on the highly corrugated iodine monolayer.

6. I-Ag(111) after DITP deposition and high temperature annealing

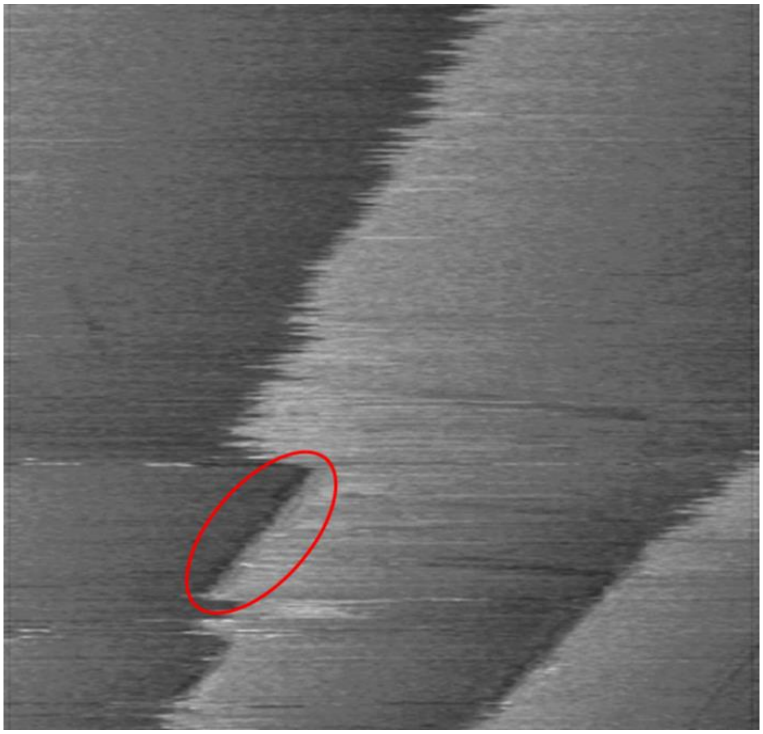

Figure S5. STM image acquired after conventional deposition of intact DITP molecules onto I-Ag(111) and subsequent annealing to 475 K (image parameters: 75×75 nm^2, 98 pA, 1.00 V); Interestingly, sections of step-edges appear straight (example marked by the red circle), whereas step-edges on pristine I-Ag(111) surfaces normally appear fuzzy (as also seen here). This may hint toward the presence of short PPP oligomers on the step-edges that have formed upon surface-induced deiodination of DITP at sparse active sites.

7. Conventional Deposition of DITP onto incompletely iodine passivated Au(111)

Noteworthy, incomplete passivation of both I-Ag(111) or I-Au(111) surfaces by too low iodine exposures resulted in mildly reactive surfaces. Deposition of intact DITP onto incompletely passivated I-Au(111) and subsequent heating to 375 K afforded the complex mixture of intact molecules as well as both terphenyl and sexiphenyl biradicals shown in Figure S7. We propose that remaining iodine vacancies in the monolayer act as active sites for deiodination.[8]

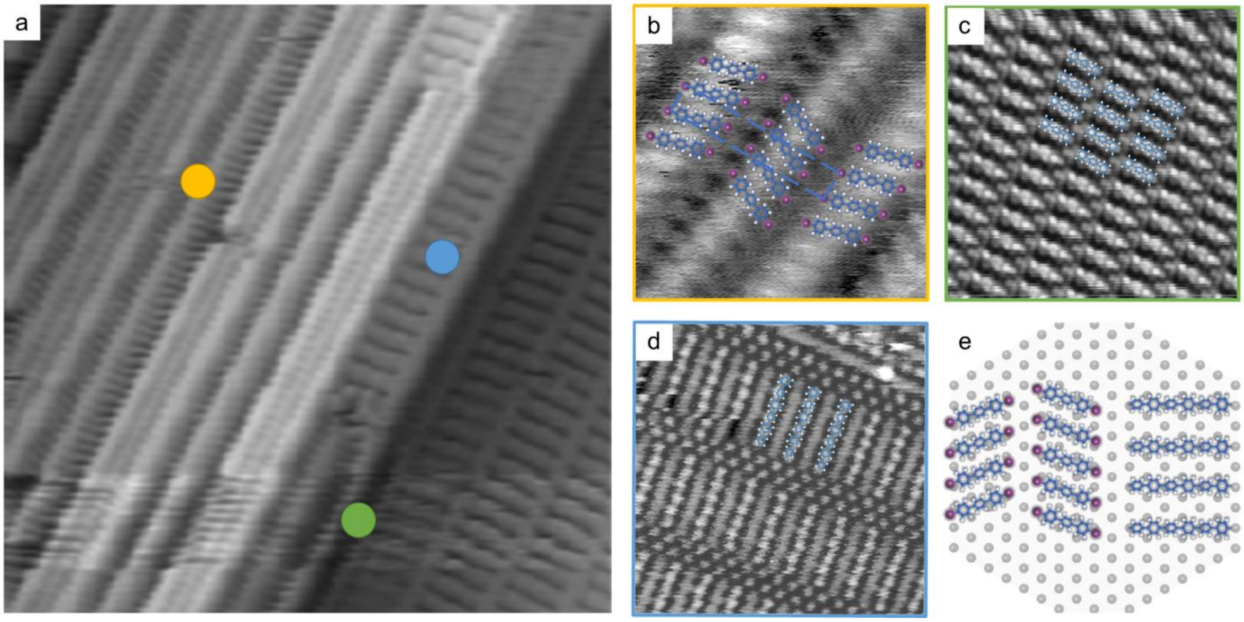

Figure S6. Conventional deposition of intact DITP molecules onto an incompletely iodinated I-Au(111) held at room temperature. **a)** STM image acquired after subsequent heating to 375 K showing different phases (image parameters: 30×30 nm^2, 217 pA, -1.20 V); These phases were previously observed either for deposition of **b)** intact DITP molecules with a Knudsen cell or **c, d)** biradicals with the RDS: lamella polymorph (marked in orange; image in b) as in Figure S3b); self-assemblies of c) terphenyl biradicals (marked in green, image as in Figure 3b of the main manuscript) or d) sexiphenyl biradicals (marked in blue image as in Figure 1b of the main manuscript); **e)** tentative model from neighboring domains of intact DITP molecules and sexiphenyl biradicals.

8. Possible non-linear junctions

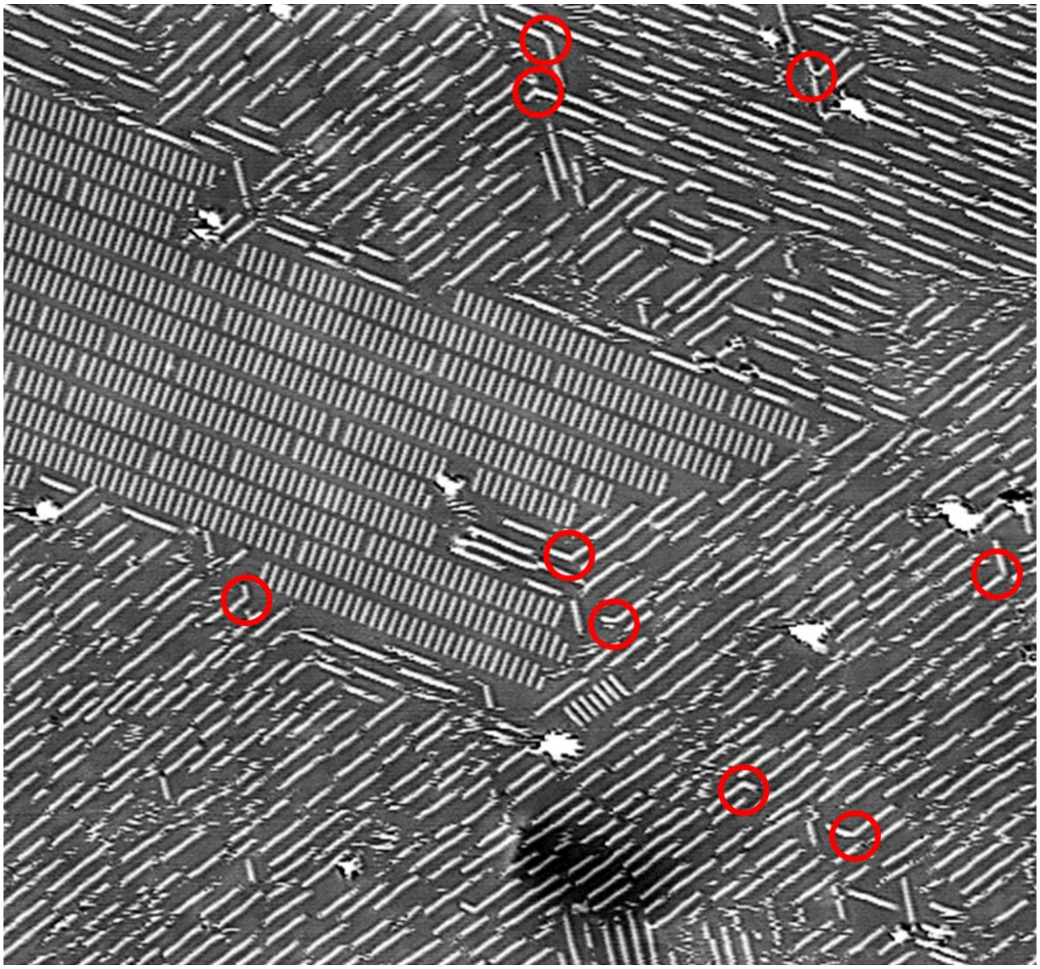

Figure S7. RDS deposition of biradicals from DITP precursors onto I-Au(111) held at room temperature and subsequently heated at 375 K. This STM image was acquired at room temperature (image parameters: 120×120 nm^2, 42 pA, 1.76 V); The red circles highlight non-linear junctions, where segments of PPP wires form angles of 60° and 120°, respectively. In some cases, these may just result from the encounter of distinct, but differently aligned PPP wires. In other cases, these may actually be kinks in a coherent wire. Nevertheless, the proportion of non-linear junctions is extremely small.

9. References

[1] D. Nečas, P. Klapetek, *Open Phys.* **2012**, *10*, 181-188.

[2] a) J. Eichhorn, D. Nieckarz, O. Ochs, D. Samanta, M. Schmittel, P. J. Szabelski, M. Lackinger, *ACS Nano* **2014**, *8*, 7880-7889; b) S. Schlögl, W. M. Heckl, M. Lackinger, *Surf. Sci.* **2012**, *606*, 999-1004.

[3] a) U. Bardi, G. Rovida, *Surf. Sci.* **1983**, *128*, 145-168; b) T. Yamada, K. Ogaki, S. Okubo, K. Itaya, *Surf. Sci.* **1996**, *369*, 321-335.

[4] G. Cavallo, P. Metrangolo, R. Milani, T. Pilati, A. Priimagi, G. Resnati, G. Terraneo, *Chem. Rev.* **2016**, *116*, 2478-2601.

[5] R. Gutzler, C. Fu, A. Dadvand, Y. Hua, J. M. MacLeod, F. Rosei, D. F. Perepichka, *Nanoscale* **2012**, *4*, 5965.

[6] F. De Marchi, G. Galeotti, M. Simenas, M. Gallagher, E. Hamzehpoor, O. MacLean, R. R. Malakalapalli, Y. Chen, D. Dettmann, G. Contini, E. Tornau, M. Ebrahimi, D. Perepichka, F. Rosei, *Nanoscale* **2019**.

[7] T. J. Mooibroek, P. Gamez, *CrystEngComm* **2013**, *15*, 1802-1805.

[8] A. Rastgoo-Lahrood, J. Björk, M. Lischka, J. Eichhorn, S. Kloft, M. Fritton, T. Strunskus, D. Samanta, M. Schmittel, W. M. Heckl, M. Lackinger, *Angew. Chem. Int. Ed.* **2016**, *55*, 7650-7654.
